# Non-Hodgkin lymphoma affecting the tongue: unusual intra-oral location

**DOI:** 10.1186/1758-3284-3-1

**Published:** 2011-01-04

**Authors:** Francesco Inchingolo, Marco Tatullo, Fabio M Abenavoli, Massimo Marrelli, Alessio D Inchingolo, Angelo M Inchingolo, Gianna Dipalma

**Affiliations:** 1 Department of dental sciences and surgery, general hospital, Bari, Italy; 2 Department of medical biochemistry, medical biology and physics, university of Bari, Bari, Italy; 3 Department of head and neck diseases, hospital "Fatebenefratelli", Rome, Italy; 4 Department of maxillofacial surgery, Calabrodental, Crotone, Italy; 5 Department of dental sciences and surgery, university of Milan, Milan, Italy

## Abstract

**Introduction:**

The expression non Hodgkin lymphoma is used to cover a wide group of lymphoid neoplasias unrelated to Hodgkin's disease, due to the huge histological variety and the tendency to affect organs and tissues that does not physiologically contain lymphoid cells.

The intraoral location is not frequent (3 - 5 percent of cases) and the initial manifestations of the disease rarely take place here.

**Case presentation:**

We describe the case of a 73 years old Italian caucasian male who came to our attention with a tongue lesion. The clinical manifestation was macroglossia and bleeding, probably deriving from the tongue-bite injuries.

The patient had been complaining of dyspnea for 48 hours.

**Conclusion:**

A tongue affected by non-Hodgkin's lymphoma rarely occurs. In spite of this, this possibility should always be considered for the differential diagnosis of benign and malignant lesions affecting such area.

A rapid diagnostic assessment, together with an adequate histopathologic verification, are indeed essential to improve the management and the prognosis of this disease.

## Introduction

Lymphomas represent the third most frequent neoplasia on a worldwide scale and constitute 3% of malignant tumors. Their prevalence progressively grows at the annual rate of 3%.

The WHO classification of tumors, which result from the hematopoietic tissues and the lymphoid tissues, allows to distinguish lymphoid neoplasias, according to the cell line and the differentiation, into:

• Hodgkin's lymphoma;

• B-cell neoplasia;

• peripheral B-cell neoplasia;

• B-cell proliferations of uncertain malignant potential;

• T-cell neoplasia;

• Peripheral T/NK-cell neoplasia;

• T-cell proliferations of uncertain malignant potential [1-3].

The main histopathologic feature of Hodgkin's lymphoma is the presence of Reed-Stenberg cells (binucleated or multinucleated, with big and clear nuclei and intensely colored "owl's eyes" nucleoli).

The above-mentioned Reed-Stenberg cells are not found in the so-called non-Hodgkin's lymphoma, a category that include all the other histopathologic entities that are not related to Hodgkin's disease [4]. The considerable histological variety of non-Hodgkin's lymphomas involves significant classification problems, although from a morphological point of view, we can identify two main forms of NHL:

• *Nodular (or follicular) forms*, characterized by a regular nodular pattern. They affect the whole lymph node or the extranodal area;

• *Widespread forms*, in which neoplastic cells are uniformly distributed on the affected tissue [5].

From a clinical point of view and in almost all cases, Hodgkin's lymphoma is a nodular lesion that rarely involves the extranodal areas, whereas NHL frequently have an extranodal onset.

In addition, whereas Hodgkin's disease spreads in the nodal groups in a *contiguous *fashion, in NHL the nodal evolution proceeds *randomly*; this means that it does not proceed in a contiguous fashion, but is instead unforeseeable (obviously, this aspect has important repercussions on the therapeutic protocol).

It is interesting to notice a geographic variability between the North and the South of Italy, with a ratio of 2:1 in favor of northern regions.

Apart from age, there are several risk factors associated to NHL:

1) Primary or acquired immunodeficiency [6,7];

2) Autoimmune diseases (Sjögren's Syndrome, LES, AR, Coeliac disease) [6], especially if treated with immunosuppressant drugs[8,9];

3) Infective agents, such as:

- Herpetic viruses (EBV, associated to the African form of Burkitt's lymphoma [8,9], HHV8, associated to Kaposi's Sarcoma[8] and found in some forms of NHL in HIV-positive subjects);

- HCV, whose association with non-Hodgkin's lymphoma is considered to be very high in several European countries with a high prevalence of this infection) [6-9];

- H. Pylori, associated to peptic ulcer and gastric MALT lymphoma [6-9];

4) Professional exposure to noxious chemical agents [6-8];

5) Hereditary factors [6-8].

Chromosomal translocations play a crucial role in the pathogenesis of NHL, determining oncogenes activation or the inactivation of oncosuppressor genes, with the consequent malfunction of the mechanism of genomic rearrangement in the lymphoid cells [6].

The primary sites most frequently affected are:

- Sovraclevear and laterocervical lymph nodes (regarding nodal sites)

- Extranodal sites (20-30% of cases) such as Waldeyer's ring, the gastroenteric tract, the skin and the subcutaneous tissue.

In the successive stages, there is the frequent involvement of the bone marrow and spleen, causing splenomegaly, which is almost constant in the immunoblastic form. A severe splenomegaly usually indicates a leukemic progression [10].

At the level of the oral cavity, they can originate from the lymphoid tissue associated to mucosa (Waldeyer's ring) or can be infiltrations of non-lymphoid tissue.

The most affected sites are tonsils (55% of oral cases), palate (30% of cases), genial mucosa (2% of cases). There are, instead, sporadic manifestations affecting the tongue (2% of cases), the buccal floor (2% of cases) and the retromolar trigone (2% of cases) [5].

From a clinical point of view, they manifest themselves with an asymptomatic tumefaction, often associated to mucosa ulceration.

The most common type in the head-neck area is the *big cells *type [6,11].

## Case presentation

A 73 years old caucasian male patient came to the authors' attention while they were providing an emergency first aid service; the patient presented hypertension on treatment with ACE-inhibitors, chronic atrial fibrillation on treatment with oral anticoagulants, and diabetes mellitus on treatment with biguanides: the clinical manifestation was bleeding, probably deriving from the tongue-bite injuries.

The patient had been complaining of dyspnea for 48 hours, caused by macroglossia.

The clinical documentation that the patient provided, allowed the Authors to identify the following clinical history: in the previous month, following the progressive volumetric increase of the tongue, he had undergone neck CT scan with contrast agents, that showed a volumetric increase of the tongue, which was occupied by a solid lesion of around 6 cm, contiguous to the muscles of the oral floor. In the submandibular and laterocervical area, there were also some lymph nodes of 15 mm in diameter at the most.

The patient referred that he had previously undergone surgery, which was planned by another clinician, as inferred from the discharge form.

In general anesthesia together with an infiltration of local anesthetic, the clinician performed a losangic biopsy (4 × 4 cm), an extemporary histological exam and suture: the intraoperatory examination showed extensive lymphoid proliferation, composed of cells of big and middle size, also affecting the skeletal muscle. The medical report indicated an extensive NHL.

In order to make an accurate diagnosis, the patient received:

- CT scans of the thorax, abdomen and pelvis, reporting enlarged lymph nodes of 10 mm in diameter in the subcarinal area and in the right pulmonary hilum;

- Hematologic consultation and osteomedullary biopsy.

After about 30 days since the first surgical removal of the tongue lesion, the patient came back at our clinic complaining severe dyspneic symptoms. Therefore the patient was immediately received by the Authors, who suspected a possible relapse of the primary lesion.

**Figure 1:**
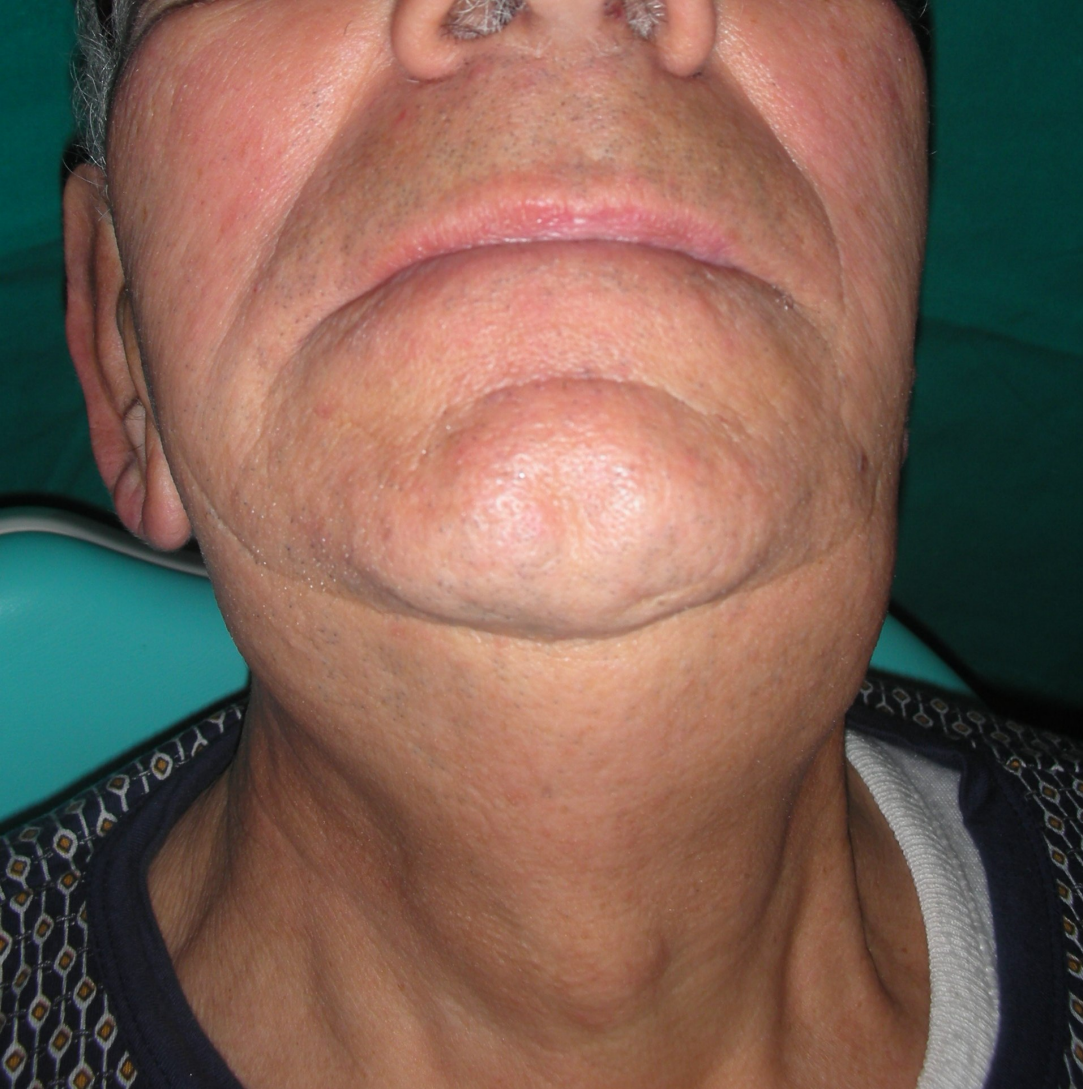
**Hard tumefaction of a normal color in the left submandibular region**.

**Figure 2:**
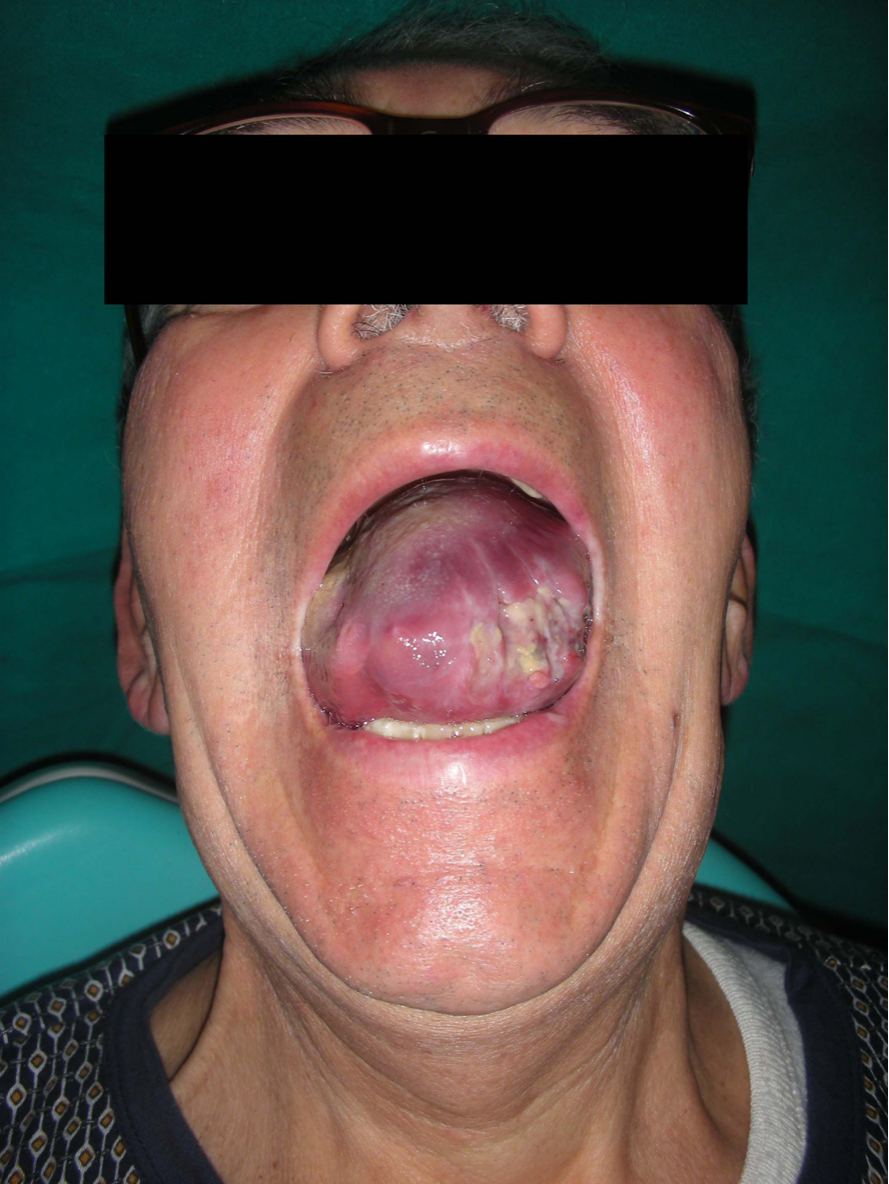
**Neck tumefaction affecting the same tongue's zone side**.

The extraoral exam revealed a hard tumefaction of a normal color in the left submandibular region (Figures 1 &2).

**Figure 3:**
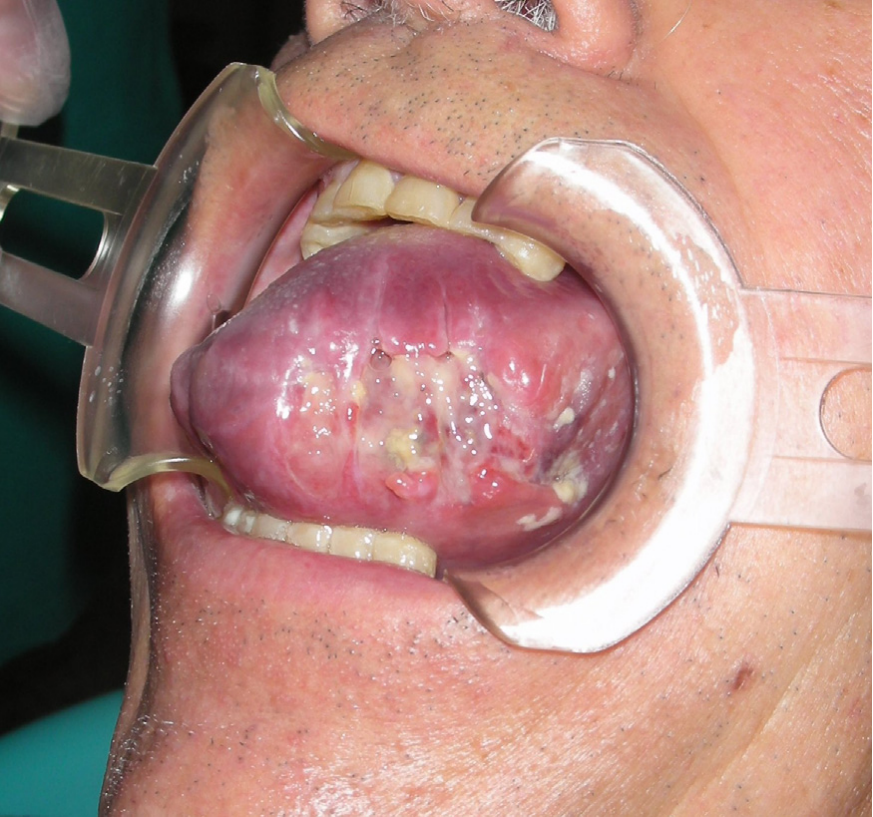
**Considerable volumetric increase of the tongue**.

**Figure 4:**
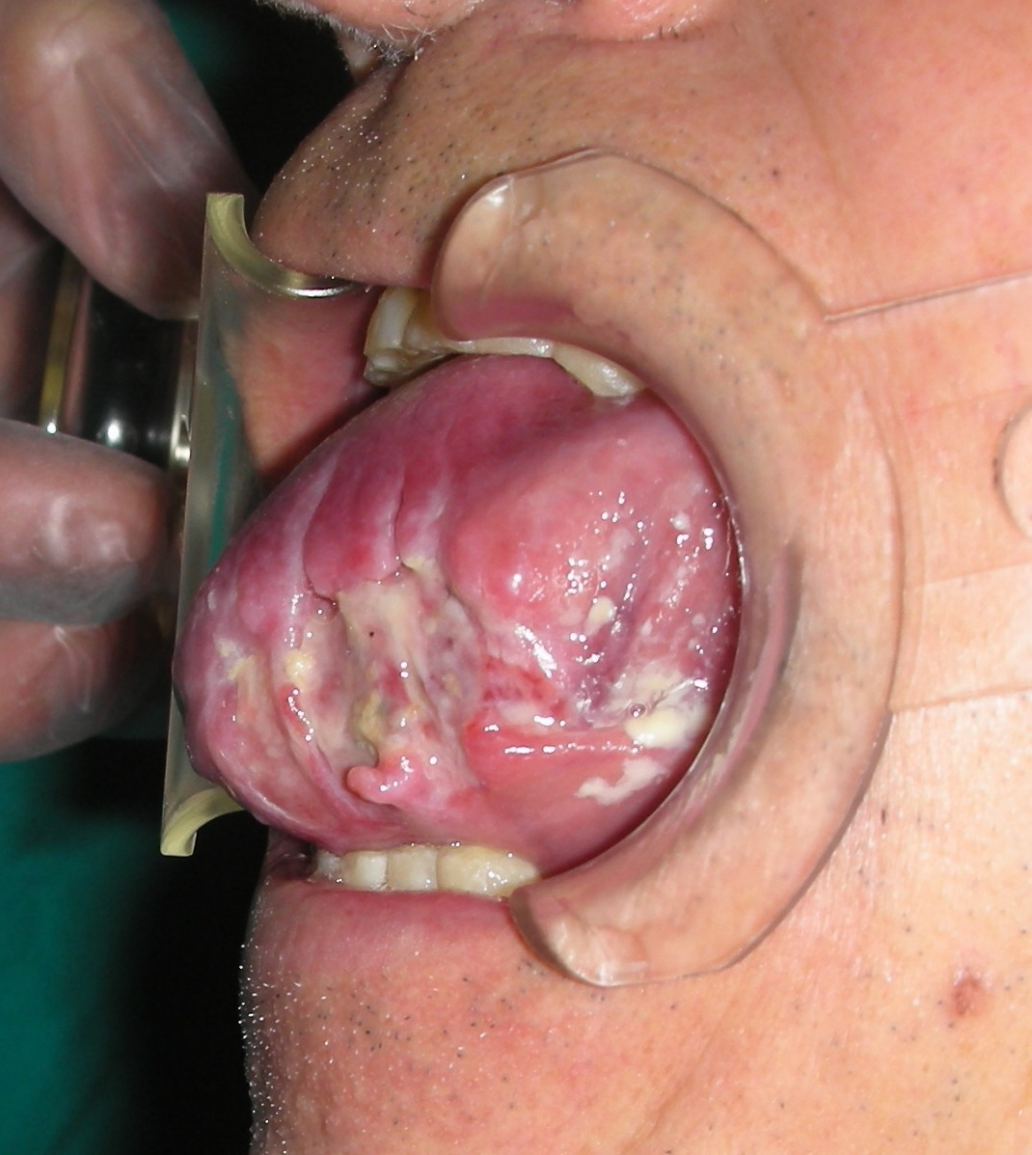
**A picture showing the entire involvement of the emi-tongue on the left side**.

The intraoral exam revealed a considerable volumetric increase of the tongue (Figures 3 &4), which was of a red-violet color, with well-delimited necrotic-ulcerative areas at the level of the left margin and left region, and with bleeding probably caused by the tongue-bite injury.

The tongue was painful and woody to palpation.

72 hours later, a fiber-scopic examination of the larynx showed *ab-extrinsic *compression of the posterior wall of the trachea, with reduction of the tracheal lumen; after the worsening of breathing, other colleagues performed an emergency inferior tracheostomy and provided medical advice.

## Discussion

The expression non-Hodgkin's lymphoma is used to cover a wide group of lymphoid neoplasias unrelated to Hodgkin's disease, due to the huge histological variety and the tendency to affect organs and tissues that does not physiologically contain lymphoid cells.

The extranodal site is the primary site of disease in 20-30% of cases; in particular, the most affected areas are the gastroenteric tract and the head-neck area (in descending order) [12].

The intra-oral location is not frequent (3-5% of cases) and the initial manifestations of the disease rarely take place here [13].

The most affected sites are tonsils (55% of oral cases), palate (30% of cases), genial mucosa (2% of cases). There are, instead, sporadic manifestations affecting the tongue (2% of cases), the buccal floor (2% of cases) and the retromolar trigone (2% of cases) [5].

In addition to the small number of cases reported in literature, this disease is also characterized by few signs and symptoms, and a frequent and dangerous resemblance to benign orodental conditions. Obviously, these features are potentially responsible for an incorrect or late diagnosis and an underestimation of the pathology, thus drastically worsening the prognosis.

The prognosis, in fact, strictly depends on the histologic type and is undoubtedly correlated to a rapid diagnosis.

## Conclusion

A tongue affected by non-Hodgkin's lymphoma rarely occurs [13].

In spite of this, this possibility should always be considered for the differential diagnosis of benign and malignant lesions affecting such area. A rapid diagnostic assessment, together with an adequate histopathologic verification, are indeed essential to improve the management and the prognosis of this disease.

## Consent Statement

Written informed consent was obtained from the patient for publication of this case report and accompanying images. A copy of the written consent is available for review by the Editor-in-Chief of this journal.

## Competing interests

The authors declare that they have no competing interests.

## Authors' contributions

FI: performed the emergency treatment of this case. MT: drafted the manuscript and reviewed the literature. FMA: participated in the follow-up of this patient. MM: participated in the design of this case study and in the follow-up of this patient. ADI: revised the literature sources. AMI: documented this case report with digital pictures. GD: participated in the follow-up of this patient. All the authors read and approved the final manuscript
